# Smartphones and Video Cameras: Future Methods for Blood Pressure Measurement

**DOI:** 10.3389/fdgth.2021.770096

**Published:** 2021-11-12

**Authors:** Joe Steinman, Andrew Barszczyk, Hong-Shuo Sun, Kang Lee, Zhong-Ping Feng

**Affiliations:** ^1^Department of Physiology, University of Toronto, Toronto, ON, Canada; ^2^Dr. Eric Jackman Institute of Child Study, University of Toronto, Toronto, ON, Canada; ^3^Department of Surgery, University of Toronto, Toronto, ON, Canada

**Keywords:** blood pressure, imaging, physiology, hemodynamics, cardiovascular digital health

## Abstract

Regular blood pressure (BP) monitoring enables earlier detection of hypertension and reduces cardiovascular disease. Cuff-based BP measurements require equipment that is inconvenient for some individuals and deters regular home-based monitoring. Since smartphones contain sensors such as video cameras that detect arterial pulsations, they could also be used to assess cardiovascular health. Researchers have developed a variety of image processing and machine learning techniques for predicting BP *via* smartphone or video camera. This review highlights research behind smartphone and video camera methods for measuring BP. These methods may in future be used at home or in clinics, but must be tested over a larger range of BP and lighting conditions. The review concludes with a discussion of the advantages of the various techniques, their potential clinical applications, and future directions and challenges. Video cameras may potentially measure multiple cardiovascular metrics including and beyond BP, reducing the risk of cardiovascular disease.

## Introduction

Blood pressure (BP) measurement is necessary in determining an individual's risk for cardiovascular disease and the need for early treatment. Early detection and treatment of BP may delay or prevent conditions related to high BP, such as stroke. This is particularly important in the Covid era, where there has been an increase in the number of virtual consultations with patients (1). Digital or at-home methods where individuals accurately and easily determine BP may improve population health, while minimizing hospital visits.

Methods for measuring BP at home or in the clinic are commonly cuff-based. Cuff-based systems are automated; however, they present difficulty in portability outside the home. Many individuals find application of the cuff awkward, inconvenient, and uncomfortable. This limits the number of daily BP measurements. Since BP varies according to time, season, amount of sleep, and activity, a single measurement over the course of a day, or every few days, does not provide an accurate assessment of cardiovascular changes and BP variation in an individual (2).

Smartphones could serve as alternatives to the cuff. Many individuals possess smartphones and operate their features with ease. Phones are embedded with cameras, microphones, light emitters, and force sensors that can be used to obtain a cardiovascular pulse signal, and ultimately predict BP. Due to their size, they overcome issues of portability, discomfort, or inconvenience.

Methods that utilize video cameras to predict BP are continually undergoing research and development for improved accuracy. Most smartphone techniques utilize the video camera to extract the photoplethysmography (PPG) signal from light reflected from the skin. Due to complexity in relating the PPG signal to BP, methodologies have been developed to determine BP from video PPG. This includes image processing to extract blood flow, machine learning algorithms for calculating BP, and incorporation of smartphone features such as the microphone or force sensors. Mathematical models may be applied to separate hemoglobin signals from melanin and light, and the shape of the pulse or different arrival times of the pulse used to predict BP (3).

This paper focuses on currently published video camera and smartphone methods for BP measurement, highlighting research efforts and experiments from a variety of groups. The two categories of smartphone/video-camera BP measurements, contact and non-contact, are covered. Present use of these techniques is discussed, together with their clinical applicability. We conclude by discussing the role of video cameras in health and BP monitoring. This paper provides an in-depth review of BP-video camera measurement technologies, including their accuracy, image processing methodologies, and machine learning algorithms used for predicting BP. This will provide a resource for researchers in this field to compare the advantages/disadvantages and technical details of various published methods.

Video cameras and smartphones could measure BP non-invasively. Through simultaneously measuring additional cardiovascular properties such as heart rate or blood oxygenation, video camera technologies may provide continuous monitoring of multiple cardiovascular properties from an individual's home.

## Non-invasive Smartphone Contact Measurements of Blood Pressure

**Figure 1:**
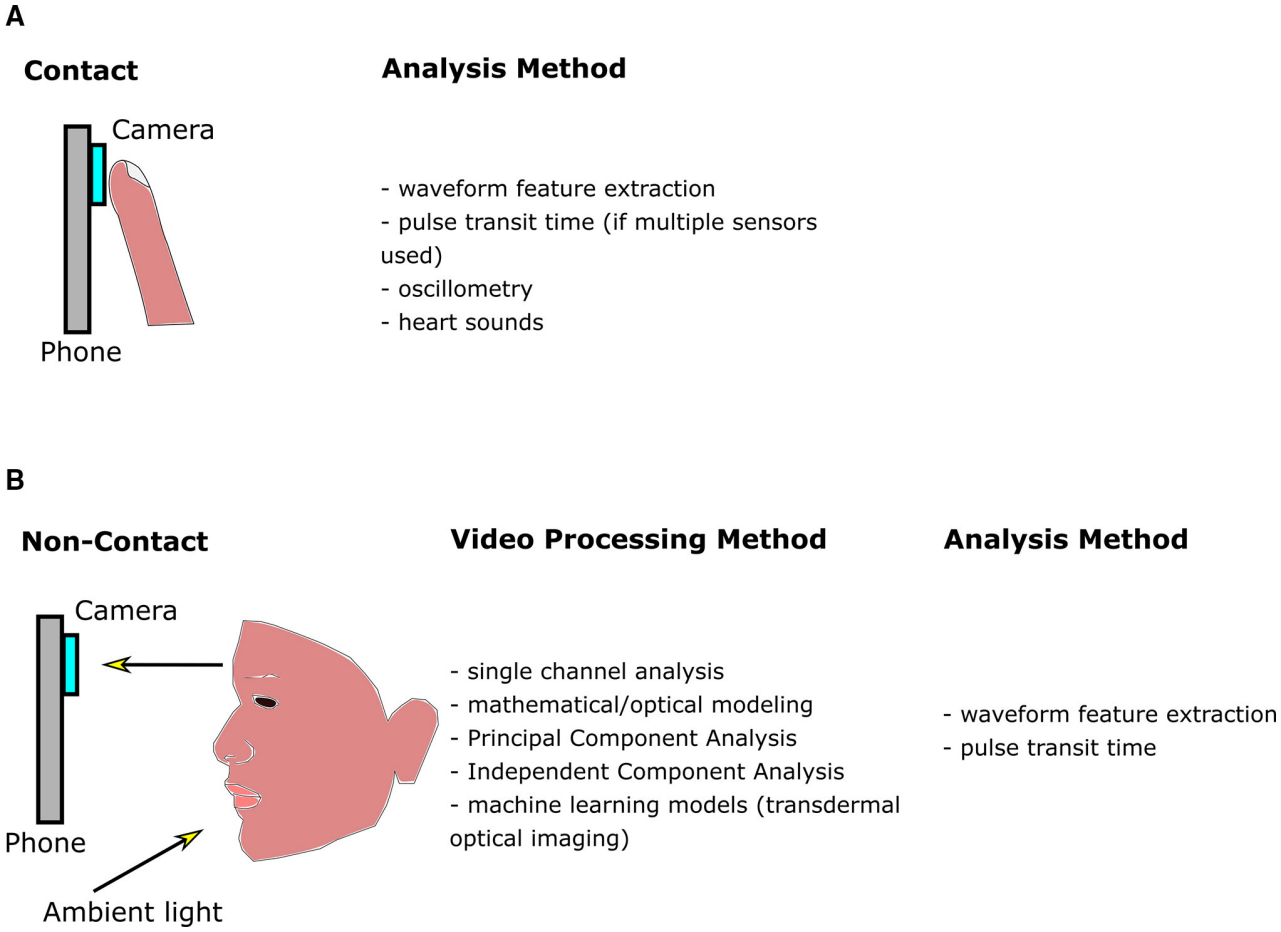
Diagram outlining methods for smartphone blood pressure (BP) estimation. **(A)** Contact methods, which require pressing the finger against the phone camera to obtain a finger blood volume pulse. Pixels within the video are averaged in each frame. The signal may be further processed and filtered, producing a waveform as a function of time. Features are extracted from the waveform and input into machine learning algorithms to calculate BP. Pulse transit time (PTT) may also be correlated with BP, however multiple sensors are required. **(B)** Non-contact methods utilize ambient light reflected from the face. The resulting video is processed to enhance the signal-to-noise ratio of the hemoglobin signal, whose features are be input into a machine learning algorithm to calculate BP (similar to contact methods). Since multiple facial regions or body parts may be imaged simultaneously, PTT may be estimated using only a single camera by estimating the difference between pulse arrival at different body regions.

Contact methods typically involve pressing the fingertip against the rear camera to acquire a PPG signal. The theory of reflection mode PPG in video cameras is initially discussed. This is followed by a description of the three categories of contact-based BP measurements (see Table 1 and Figure 1): (1) Oscillometry; (2) Analysis of pulse waveform features; (3) and Pulse transit time (PTT), calculated as the time delay between two PPG waveforms at different arterial sites.

**Table 1:** Contact methods for smartphone blood pressure (BP) measurement.

**Publications**	**Number of subjects in study and additional experimental details**	**Accuracy of method**	**Analysis/processing method**
Chandrasekaran et al. (4)	5 subjects -2 phones (1 camera, 1 microphone) OR single phone camera with external microphone	*Still with single mobile:*SP: 98.59% accuracy DP: 97.96% accuracy*Movement with single mobile:*SP: 97.45% accuracy DP: 97.63% accuracy - mean accuracy values calculated by Steinman et al.: original data in paper provided individual but overall mean values	Pulse Transit Time
Lamonaca et al. (5)	5 experiments	Max error in SP: 11 mHg Max error in DP: 12 mmHg	Waveform Analysis
Visvanathan et al. (6)	17 subjects	*Linear regression:*SP: 98.7% detection accuracy DP: 99.7% detection accuracy *SVM:* SP: 100% detection accuracy DP: 99.29% detection accuracy * BP values divided into bins	Waveform Analysis
Visvanathan et al. (7)	156 subjects	SP: 98.81% detection accuracy (cross validation) DP: 98.21% detection accuracy (cross validation) * BP values divided into bins	Waveform Analysis
Banerjee et al. (8)	23 subjects (15 training, 8 testing)	SP: 4 ± 2 mmHg (MAE ± std) DP: 4 ± 2 mmHg (MAE ± std) - values calculated by Steinman et al.; original data in paper only provided for individuals but not averaged	Waveform Analysis
Liu et al. (9)	12 subjects - 2 cameras (1 for fingertip, other for forehead temple)	- correlation 0.86 ± 0.06 between established PTT and OFP * OFP is the time interval between minimum PPG signal from temple and maximum of PPG signal from fingertip	Pulse Transit Time
Peng et al. (10)	32 subjects stethoscope attached to phone	SP: 4.339 ± 6.121 (MAE ± std) DP: 3.171 ± 4.471 (MAE ± std) MP: 3.480 ± 4.961 (MAE ± std)	Heart Sounds Only
Junior et al. (11) Junior et al. (12)	3 subjects - heart sounds and camera	Mean percent error in automated vs. manual calculation of PTT: 2.53% (maximum 3.00%)	Pulse Transit Time
Gao et al. (13)	65 subjects	SP: 5.1 ± 4.3 mmHg (ME ± std) DP: 4.6 ± 4.3 mmHg (ME ± std)	Waveform Analysis
Plante et al. (14)	85 subjects - heart sounds and camera	SP: 12.4 ± 10.5 mmHg (MAE ± std) DP: 10.1 ± 8.1 mmHg (MAE ± std)	Pulse Transit Time
Datta et al. (15)	118 subjects (68 training from oximeter PPG; 50 smartphone PPG for testing)	SP: Mean absolute percentage difference 7.4% Correlation 0.57 with ground truth SBP DP: Mean absolute percentage difference 9.1 % Correlation 0.40 with ground truth DBP	Waveform Analysis
Chandrasekhar et al. (16)	32 subjects (35 subjects originally) - special case used to measure PPG and applied force	SP: 3.3 ± 8.8 mmHg (ME ± std)DP: −5.6 ± 77 mmHg (ME ± std)	Oscillometry
Chandrasekhar et al. (17)	18 subjects (20 subjects originally) - phone camera, iPhoneX 3D touch feature to measure applied force	SP: −4.0 ± 11.4 mmHg (ME ± std) DP: −9.4 ± 9.7 mmHg (ME ± std)	Oscillometry
Dey et al. (18)	205 subjects (160 training, 45 testing)	SP: 6.9 ± 9.0 mmHg (MAE ± std) DP: 5.0 ± 6.1 mmHg (MAE ± std)	Waveform Analysis
Matsumara et al. (19)	13 subjects	SP: 0.67 ± 12.7 mmHg (ME ± std) DP: 0.45 ± 8.6 mmHg (ME ± std) MP: 0.49 ± 9.6 mmHg (ME ± std)	Waveform Analysis
Wang et al. (20)	7 subjects (nine subjects originally) - phone accelerometer and camera	DP: 5.2 ± 2.0 (RMSE ± std)	Pulse Transit Time
Baek et al. (21)	26 subjects - convolutional neural network without feature extraction	SP: 5.28 ± 1.80 (MAE ± std) DP: 4.92 ± 2.42 (MAE ± std)	Waveform Analysis
OptiBP, Schoettker et al. (22)	50 subjects for training (51 originally), 40 validation (50 originally)	SP: −0.7 ± 7.7 mmHg (ME ± std) DP: −0.4 ± 4.5 mmHg (ME ± std) MP: −0.6 ± 5.2 mmHg (ME ± std)	Waveform Analysis
Nemcova et al. (23)	22 subjects - heart sounds and camera	SP: −0.2 ± 6.7 mmHg (ME ± std) DP: −0.07 ± 8.8 mmHg (ME ± std)	Pulse Transit Time
Tabei et al. (24)	6 subjects - cameras from 2 smartphones	SP: 2.07 ± 2.06 mmHg (MAE ± std) DP: 2.12 ± 1.85 mmHg (MAE ± std)	Pulse Transit Time
Preventicus (app)	Raichle et al. (25): 32 pregnant women Dörr et al. (26): 965 subjects (1,036 subjects originally)	Raichle 2018: SP: 5.0 ± 14.5 mmHg (ME ± std) Dörr 2021: SP: −0.41– ± 16.52 mmHg (ME ± std)	Waveform Analysis

*ME, mean error; MAE, mean absolute error; SP, systolic pressure; DP, diastolic pressure; MP, mean pressure; PTT, pulse transit time; PPG, photoplethysmography*.

### Reflection Photoplethysmography and Smartphones: An Introduction

The heart generates pulsatile flow, causing blood vessels in the skin to expand and contract. Light absorption by hemoglobin in the blood is maximized when the vessel is fully expanded during systole and minimized during diastole. In reflection mode PPG, light reflected from the skin is detected by a sensor or camera. The PPG waveform has an oscillating “AC” component largely due to arterial pulsation, which is superimposed on a DC component corresponding to fat and blood volume.

In 2010, the smartphone was used to obtain a PPG signal for heart rate assessment by pressing the finger against the rear camera (27). Although data from red, green, and blue color channels are obtained, the green channel is typically used for calculating physiological parameters *via* video camera methods. This is likely due to green light possessing higher absorption by hemoglobin than red, while penetrating deeper into tissue than blue (28). These techniques have been extended to estimate BP (systolic, diastolic, mean) *via* a logarithmic equation relating pressure to heart rate and pulse volume (19).

### Application of Oscillometry to Smartphones

Oscillometry techniques produce an automated digital pressure output and determine BP with limited user input. Vibrations produced through opening of the arterial wall travel through air inside the cuff, and into a transducer producing an electrical signal (29). The upper and lower envelopes of the oscillation are traced as cuff pressure varies from above systolic pressure (SP) to below diastolic pressure (DP). Algorithms then estimate the mean, systolic, and diastolic pressures from the oscillogram (plot of the oscillation amplitude vs. cuff pressure) (30, 31).

Oscillometry has been extended to smartphones. Chandrasekhar et al. (16) developed a smartphone-based device to detect varying pressure in a finger artery, similar to that of a changing cuff pressure as with cuff-based oscillometry. A case was attached to the smartphone that contained a PPG sensor overlaying a force transducer, since a sensor capable of detecting force applied by the finger was not present within the phone itself. An infrared LED illuminated the finger pressed against the PPG sensor, and the force sensor detected the varying force applied by the user. An oscillogram was generated, and finger BP related to brachial BP *via* fitting a parametric model to the oscillogram.

Most users found the technique user-friendly and learned it after 1–2 trials. However, whereas the accuracy of this technique was comparable to a finger-cuff, only approximately 60 % of BP measurements were successful with the device. More than half the failures to output a BP value were attributed to a “computation failure.” A special case was also required for this method that incorporated sensors for production of a finger oscillogram. An additional study by the same group (17) incorporated the iPhone X's built-in 3D touch force sensor and camera for PPG detection, eliminating the need for a special case.

### PPG Waveform Analysis to Predict Blood Pressure

#### Calculating Blood Pressure From Waveform Analysis

A common approach to calculating BP is to extract features from the pulse waveform related to the shape of the pulse. The user turns on the LED flash, presses their finger against the phone camera, and records a video. The resulting video can be analyzed to produce a pulsatile waveform. Features are then extracted characterizing the waveform such as pulse width, slope of initial upstroke, height, time between pulses, etc. These features are input into machine learning models, such as neural networks or regression models, thereby calculating blood pressure. Multiple features render algorithms less susceptible to data variability, and increase pressure calculation accuracy. Unlike methods such as PTT (see below), only one sensor is needed, and it is less sensitive to motion artifacts because the finger is pressed against the camera. This also produces a stronger signal than with non-contact video PPG methods (section Non-contact Video Camera Measurement of Blood Pressure).

Use of the PPG waveform to predict BP is not limited to smartphone-based PPG. As such, there is a range of machine learning methods that are used, and methods for acquiring data. For example, datasets may be publicly available, such as through the Multiparameter Intelligent Monitoring in Intensive Care MIMIC database, (32) or frequently acquired *via* pulse oximeter (33).

PPG waveform analysis faces challenges, several of which are outlined in (34). Briefly, PPG waveforms are an indirect measure of pressure. Finger sizes and pressing pressure vary considerably between subjects, affecting the PPG waveform and consequently influencing prediction accuracy. Diseases, such as anemia, reduce hemoglobin concentration and alter the relationship between blood volume and total hemoglobin. Other diseases alter the circulation and body temperature, in turn reducing the correlation between the peripheral pulse measured with PPG and BP. Nevertheless, arterial BP, and PPG signals have a high similarity in their morphology, with potential for determining whether patients are normotensive or hypertensive (35). Consequently, studies employing PPG waveform analysis *via* smartphones (as detailed below) have potential for predicting BP and diagnosing conditions such as hypertension.

Lamonaca et al. (5) trained a neural network on 15,000 PPG pulses with associated pressure from MIMIC. Features from the PPG pulses relating to length of time in portions of the cardiac cycle, systolic upstroke time, diastolic time, and cardiac period were extracted and input into the neural network algorithm for training on the database. This trained network was applied to PPG pulses acquired with a smartphone and compared to pressure measured in the arm with a cuff. Over five experiments, the maximum difference between predicted and reference systolic values was 11 and 12 mmHg between diastolic values. This is above the accuracy threshold of 5 ± 8 mmHg according to the Association for the Advancement of Medical Instrumentation.

Visvanathan et al. (6) analyzed 14 time domain features of the PPG waveform, in addition to height, weight, and age. These were input into a linear regression or support vector machine classification model to estimate BP. In 2014, a similar analysis was performed that included additional features in the time and frequency domain (7).

To reduce noise, Banerjee et al. (8) approximated the PPG signal as a sum of two Gaussian functions. The cardiovascular system was modeled as a circuit, with a peripheral resistance (R) and arterial compliance (C). SP and DP were expressed as an exponential function of R and C. Seven features from the PPG signal modeled as the sum of Gaussians were input into a neural network to calculate R and C, enabling estimation of SP and DP.

Gao et al. (13) applied a discrete wavelet transform to the PPG signal to extract periodic features. Feature selection was performed using a linear support vector machine, followed by training a non-linear support vector machine to predict BP. The mean error for DP was 4.6 ± 4.3 mmHg, and the error for SP of 5.1 ± 4.3 mmHg.

Datta et al. (15) analyzed the ratio of PPG features, systolic upstroke time, the inverse of systolic upstroke time squared, and age and body mass index (six features total). Mean absolute error values of 7.4% (systolic) and 9.1% (diastolic) were obtained. The advantage of measuring the ratio of features is to reduce dependence on use of a particular camera or smartphone. This provides an algorithm that is applicable between phones and manufacturers, and is less likely influenced by sensor or phone properties.

Dey et al. (18) incorporated 233 total features in the time and frequency domain. A mean absolute error for DP of 5.0 ± 6.1 mmHg, and 6.9 ± 9.0 mmHg for SP was calculated.

These studies used feature extraction to predict BP, which may be influenced by sensor and signal quality, and vary between studies. It is possible to eliminate feature extraction, as demonstrated by Baek et al. (21). In this study, a convolutional neural network was applied to PPG signals without feature extraction. They obtained a mean absolute error for DP of 4.92 ± 2.42 mmHg, and 5.28 ± 1.80 for SP.

Large-scale studies in patients with a range of BP values are necessary to assess the accuracy of an app or technique, since inaccuracy may be induced at higher or lower BP ranges. This could be attributable to utilizing training data largely from normotensive populations, healthy/younger individuals, or using invasive BP measures (instead of cuffs) as reference data (26). The Preventicus BP estimation algorithm overestimated SP in low BP ranges (<130 mmHg), and underestimated SP in medium BP ranges (130–160 mmHg) in pregnant women (25). In a recent study of more than 900 individuals (300 hypertensive) using the Preventicus app, overestimation of SP occurred at lower SP, and underestimation at higher SPs, with decreasing performance at higher pressures. OptiBP, a smartphone app, possessed high accuracy when tested on a range of BPs (hypotensive to hypertensive, 101 subjects total), suggesting it is more likely to be applicable to the general population than Preventicus (22). It is difficult to determine presently the reasons similar techniques (OptiBP vs. Preventicus) are not equally successful since they both follow a similar methodology (finger-pressing). The Preventicus algorithm is a combination of frequency and morphology analysis, and utilizes the knowledge that time difference between the notch and peak represent peripheral resistance and depends on BP (26). The OptiBP algorithm obtains an average waveform over multiple measurements, with less weight attributed to pulses with abnormal morphology. Derivative-based features are extracted from the pulse, and a non-linear model is used to predict BP. The final BP is determined following a calibration procedure (22).

### Pulse Transit Time (PTT): Using Signals From Multiple Locations to Estimate Blood Pressure

#### Calculating Blood Pressure From Pulse Transit Time

Waveform analysis extracts multiple pulse features, inputting them into machine learning algorithms to calculate BP. Prediction accuracy is dependent on selection of correct features based on the waveform shape, which may depend on characteristics of the sensor. Consequently, algorithms developed on one smartphone may not be transferable to a different phone, since each uses a different set of sensors. Algorithms may require further development to automatically detect subtle features in the pulses relatable to pressure, along with acquisition of large datasets for machine learning. These techniques are often limited to the fingertip (one region), whereas pulse information from multiple regions could increase accuracy of BP estimation algorithms (36).

PTT overcomes some obstacles of waveform analysis, requiring only measurement of relative arrival times of two pulses at different points in the body. PTT is inversely proportional to pulse wave velocity, which increases with BP. It is used as an indirect measure of BP, with a reduced PTT indicating elevated pressure.

PTT and pulse arrival time (PAT) are often used interchangeably. Technically, PTT is the time difference between two points in the PPG waveforms measured at different arterial sites. PAT represents the time difference between the R-peak of the electrocardiogram and a characteristic point in the PPG waveform, such as the foot. Since PTT is not in pressure units, methods using PTT to estimate pressure require calibration to relate the quantities. Accuracy of PTT-based BP measurements therefore depends on calibration quality, possibly requiring recalibration after several months. Calibration for each individual is performed by acquiring multiple pressure and PTT measurements and performing regression analysis to relate the two quantities. Multiple pressure and PTT measurements may be acquired through pressure perturbations such as exercise or changing posture (37).

Inconsistencies impact the accuracy or variability of BP recordings calculated from PTT. The characteristic points used to determine PPG pulse arrival time differ between studies, such as the foot or peak of the waveform, or the peak of the second derivative waveform (38). Either marker may be used, although effects of wave reflection from peripheral arteries are minimized if the foot-to-foot time delay between waveforms is used (37). Conditions for accurate BP calculation from PTT include assumptions of negligible contraction of vasculature *via* smooth muscle and negligible viscous effects, which induce PTT variations without affecting blood pressure; and minimal changes to arterial elasticity in response to disease or aging (37). Due to this final condition, periodic recalibration is required for chronic BP measurements, with calibration period depending on age. In a theoretical study, for a 30-year-old the calibration period to maintain accuracy (<1 mmHg error in BP calculation) is approximately 1-year, decreasing to 6-months for a 70-year-old (39). A study in 14 normotensive subjects (aged 20–36 years) suggest shorter calibration periods, where regression coefficients for calculating BP from PTT in a first test inaccurately predict blood pressure in a repeat test 6-months later (40).

#### Incorporation of PTT Into Contact-Based Smartphone Techniques

Two measurements are required to incorporate PTT into smartphones: one PPG pulse, and a PPG pulse or indicator of heartbeat. Chandrasekaran et al. (4) used two methods for calculating BP based on recording of heart sound (phonocardiogram, PCG) and finger pulse. The first method required two smartphones. One was pressed against the user's chest to record the PCG, while the other detected the PPG finger pulse. The second method was similar, except a single phone was used. A finger PPG pulse was acquired, and a customized external microphone attached to the smartphone to amplify the acoustic heart signal. Estimated BP values achieved an accuracy of approximately 95–100% when compared to a commercial BP meter. Similar techniques combining PCG and PPG are described in Junior et al. (11, 12) and Nemcova et al. (23). Such techniques have been incorporated into phone apps, such as AuraLife Instant Blood Pressure (IBP) (41). Clinical translation of the AuraLife app has not been successful, where a clinical study (85 participants, 53% with hypertension) of the app found large errors in measured pressure and low sensitivity to detecting hypertension (14).

A potential inaccuracy induced through PCG measurement is reliance of the signal on closing instead of opening of heart valves. This provides incorrect times for PTT determination since valve closure does not indicate when blood is ejected from the heart (20). Therefore, Wang et al. (20) instead investigated the phone's accelerometer to detect vibrations caused by mechanical movement of the heart (seismocardiogram, SCG). The error in DP over all subjects was 5.2 ± 2.0 mmHg (RMSE ± std). SP was not calculated since the characteristic PPG point to calculate PTT was the foot of the pulse, which measures arrival time of diastole (20).

Some studies use two cameras and define PTT as the difference in times between two characteristic points in the PPG pulses at different body locations. Liu et al. (9) assembled a prototype device with the front-camera pressed against the temple and the finger contacting the rear-camera. Tabei et al. (24) incorporated two smartphones, defining PTT as the time difference between peak locations for each fingertip PPG. SP and DP were estimated with a regression model, and compared to calculations with a reference device. Estimates demonstrated a mean absolute error for SP and DP of ~2 mmHg.

## Non-contact Video Camera Measurement of Blood Pressure

A disadvantage of contact techniques is the PPG signal dependence on finger pressing force, in contrast to non-contact video camera techniques. Non-contact techniques can simultaneously measure multiple body parts and regions of the face, which are differentially innervated by sympathetic and parasympathetic neurons. This additional information could increase accuracy of camera prediction methods, compared to more homogeneous data acquisition as per finger-pressing contact techniques (36).

However, non-contact video methods are susceptible to noise and artifacts unrelated to the hemoglobin signal. For example, a dicrotic notch in the blood volume pulse is often absent in video PPG, which could affect calculation of PTT. While some signal reflected from tissue is due to hemoglobin, other light does not pass through tissue and is reflected from the skin surface. This is termed diffuse reflection (42). Other light reflection is due to non-hemoglobin components, such as melanin. In order to predict BP most accurately, and to avoid noise effects being interpreted as part of the signal, mathematical/optical models or machine learning techniques are used to specifically extract the hemoglobin signal component. For example, the chrominance model (CHROM) yields a higher blood volume pulse SNR than techniques such as blind source separation, even under conditions of movement due to its ability to remove non-hemoglobin components such as diffuse reflection (42). In exercise (subject movement) conditions, processing data from only a single channel often does not yield a clear blood volume pulse (43).

Consequently, a number of video camera processing techniques have been developed for overcoming deficiencies of single channel analysis, using sophisticated algorithms to minimize noise effects. This section details these techniques. Nevertheless, as will also be described, studies utilizing only a single color channel for analysis are still able to produce accurate estimates of BP or strong correlations between PTT calculations and BP.

These methods are summarized in Table 2, while Table 3 compares the advantages and disadvantages of contact and non-contact techniques.

**Table 2:** Non-contact methods for smartphone/video blood pressure (BP) measurement.

**Publications**	**Number of subjects and additional experimental details**	**Accuracy of method**	**Video processing method**
Murakami et al. (44)	10 subjects	Correlation coefficient of PTT with SP: −0.879	Single-Channel Analysis
Sugita et al. (45)	20 subjects	Correlation coefficient with SP: ~ 0.6 for pulse wave indices from right hand	Single-Channel Analysis
Yoshioka et al. (46)	10 subjects	Correlation coefficient between PTT and SP: −0.879 * same study subjects as Murakami et al. (44) above	Single-Channel Analysis
Jain et al. (47)	45 subjects	SP: 3.90 ± 5.37 (MAE ± std) DP: 3.72 ± 5.08 mmHg (MAE ± std)	Principal Component Analysis
Jeong and Finkelstein (48)	7 subjects	Correlation between SP and PTT: −0.80 - correlation obtained by averaging across values for individual subjects provided in Jeong and Finkelstein (48)	Single-Channel Analysis
Secerbegovic et al. (49)	3 subjects	PTT calculated from ECG and video forehead signal: SP: 9.48 ± 7.13 mmHg (MAE ± std) MP: 4.48 ± 3.29 mmHg (MAE ± std) Correlation between PTT phase delay between forehead and palm video signals and SP:−0.6045	Independent Component Analysis
Huang et al. (50)	13 subjects	SP: 14.02 mmHg (RMSE) DP: 7.38 mmHg (RMSE)	Single-Channel Analysis
Khong et al. (51)	45 subjects	SP: 4.22 ± 3.15 mmHg (MAE ± std) DP: 3.24 ± 2.21 mmHg (MAE ± std)	Single-Channel Analysis
Patil et al. (52)	20 subjects	Morning session SP: 9.62 % (error rate) DP: 11.63 % (error rate) Afternoon session SP: 8.4 % (error rate) DP: 11.18 % (error rate)	Independent Component Analysis
Chen et al. (53)	2 subjects	SP: −2.40 % – 3.43 % (range of error compared to reference)DP: −6.88 % – 5.26 % (range of error compared to reference)	Mathematical/Optical Modeling
Fang et al. (54)	15 subjects	SP: 11.2 mmHg (RMSE) PP: 7.83 mmHg (RMSE)	Mathematical/Optical Modeling
Viejo et al. (55)	15 subjects (70 % training, 15 % validation, 15 % testing)	Correlation coefficient in testing phase between measured BP, heart rate and reference BP, heart rate: 0.71	Single-Channel Analysis
Oiwa et al. (56)	8 subjects	MP: range from 1.50 mmHg – 4.15 mmHg (MAE)	Independent Component Analysis
Shirbani et al. (57)	15 subjects	Slope from plot of PAT measured from video PPG vs. DP: −1.33 ± 1.70 ms/mmHg (mean ± standard error), p = 0.0024	Single-Channel Analysis
Adachi et al. (3)	10 subjects	Without body movement: SP:−1.0 ± 5.6 mmHg (ME ± std) With body movement: SP: −0.1 ± 12.2 mmHg (ME ± std)	Mathematical/Optical Modeling
Luo et al. (36)	1,328 subjects (70 % training, 15 % testing, 15 % validation) (data collected from 2,348 subjects originally)	SP: 0.39 ± 7.30 mmHg (ME ± std) DP: −0.20 ± 6.00 mmHg (ME ± std) PP: 0.52 ± 6.42 mmHg (ME ± std)	Transdermal Optical Imaging
Sugita et al. (45)	17 subjects (20 subjects originally)	Correlation coefficient of right palm index with SP: < −0.5	Single-Channel Analysis
Sugita, Noro, et al. (58)	5 subjects	SP: 25.7 mmHg (RMSE)	Single-Channel Analysis
Fan et al. (59)	6 subjects	SP: 8.42 ± 8.81 mmHg (MAE ± std) DP: 12.34 ± 7.10 mmHg (MAE ± std)	Mathematical/Optical Modeling
Takahashi et al. (60)	4 subjects	Correlation between SP and PTT measured only in face *via* video: −0.4543 (range from −0.7820 to −0.2900) - average value not provided in original publication; averaged here over the four individual values	Mathematical/Optical Modeling
Rong and Li (61)	189 subjects (191 subjects originally; 70 % training; 30 % testing)	SP: 9.97, 2.1 ± 3.35 mmHg (MAE, ME ± std) DP: 7.59, 0.79 ± 2.58 mmHg (MAE, ME ± std) - showing here result obtained with the machine learning method that produces the smallest MAE	Single-Channel Analysis

*ME, mean error; MAE, mean absolute error; RMSE, root mean square error; SP, systolic pressure; DP, diastolic pressure; MP, mean pressure; PTT, pulse transit time; PPG, photoplethysmography; PP, pulse pressure; PAT, pulse arrival time*.

**Table 3:** Comparison of contact and non-contact BP measurement techniques.

	**Advantages of techniques**	**Disadvantages of techniques**	**Comments on specific contact/non-contact techniques**
**Contact**	- higher signal achievable compared to non-contact due to proximity of finger to sensor and LED - reduced sensitivity to subject motion, since the finger is pressed against the camera - less sensitive to external lighting conditions than non-contact methods	- signal may depend on finger pressing force - may require multiple sensors, such as microphone, in addition to camera - limited to certain regions of the body, such as the finger, whereas the face includes pulse information for predicting BP - may depend on height of hand relative to heart	Oscillometry - convenient, easy method to learn - may require a special case to sense applied pressure Waveform Analysis - prediction accuracy dependent on size of training data, extent to which training data reflects the characteristics of the population, and features extracted for input into machine learning algorithms Pulse Transit Time - easy, efficient measure that correlates with BP - data may require calibration every 6-months to 1-year, depending on age and health conditions of subject - multiple sensors required in the case of contact methods Heart Sounds Only - may require attachment of a stethoscope to smartphone to amplify heart sounds
**Non-contact**	- may image multiple regions simultaneously without additional equipment/sensors - signal does not depend on pressing force, yielding more consistency across subjects or between trials - acquisition of blood pressure through ‘selfie’ or short video	- sensitive to lighting conditions, angle of camera with face, and distance of camera from face - sensitivity to body and surface skin movement - relatively weak signal, since often the camera is held a distance from the face and ambient light is used as the light source	Single-Channel Analysis - usually green channel analyzed, followed by application of PTT or waveform analysis - susceptible to skin inhomogeneities, melanin, lighting conditions Transdermal Optical Imaging - use of machine learning to extract hemoglobin signal - applied in study of over 1,300 individuals to predict BP Mathematical/Optical Modeling - account for light reflectance from skin surface, skin movement, melanin, and lighting. They may therefore be potentially applied to a range of real-life conditions outside the laboratory. Independent Component Analysis - assumes each channel contains a hemoglobin component - component with the strongest signal at the heart rate selected as the pulse component Principal Component Analysis - may be used to determine which components of the video signal are attributable to hemoglobin pulsation - can reduce data redundancy for optimal performance of machine learning algorithms

### Single Channel Video Analysis

PPG signal for heart and respiratory rate calculation was detected from facial videos in 2008 by averaging pixel signal over the green channel (28). Machine learning techniques, such as neural networks, can use features from the filtered, averaged signal to predict BP (55, 61).

An advantage of video techniques is multiple regions may be imaged simultaneously, enabling calculation of PTT using only a single sensor (camera). This was demonstrated by Jeong and Finkelstein, (48) who found a strong correlation between SP and PTT through videos acquired of the face and hand. Their study used high speed video recording (420 fps) to capture 1-min videos between/before/after exercise protocols, with the subject seated and hand on a table. Other studies utilizing PTT to estimate BP from single channel processing include: Yoshioka et al. (46), Huang et al. (50), Khong et al. (51), Murakami et al. (44) and Shirbani et al. (57). As an alternative to PTT, it may be possible to capture videos of hands at different heights, relating the difference in pulse amplitudes between the hands to BP (58). Others use phase difference between PPG waveforms as a surrogate for time delay, which has a higher correlation with SP compared to time delay methods (62). Phase difference, however, is distorted by skin inhomogeneities and may not provide a truly accurate measure of PTT (63).

A disadvantage to requiring two pulse measurement locations, as in Jeong and Finkelstein (48), is PTT varies according to the distance between face and hand or body parts selected for analysis. This will alter BP prediction when a subject adjusts their posture. To overcome potential variability in measuring PTT in different regions, Sugita et al. (45) calculated time difference (T_BH_) between minimum values in a band-pass filtered waveform from video of the palm, and the raw waveform from the palm. They demonstrated smoothing the PPG waveform causes a phase change indicating heart-rate variability, with T_BH_ indicating the degree of distortion of the PPG waveform. T_BH_ showed a similarly strong correlation with SP as the difference between arrival times of waveforms in the palm and forehead. This suggests T_BH_ has similar accuracy as PTT in calculating BP, but should be more applicable to situations where body movement occurs, such as exercise.

### Processing Video Data to Extract Hemoglobin Information

Single-channel (typically green channel) data contains hemoglobin information. The overall channel signal, however, is affected by melanin content (skin tone), lighting, and subject movement. This may be controlled in experimental situations through consistent and bright lighting, and limiting subject movement. In “real world” environments, there are a variety of skin tones, background light, and subject motion. In these situations, analysis of raw or filtered green channel data risks acquisition of a distorted or altered waveform not accounted for in algorithms relating waveform shape to BP. As detailed below, video frames may be processed using machine learning or mathematical algorithms to extract hemoglobin-dependent features of the signal that are then used to estimate BP.

#### Transdermal Optical Imaging (TOI)

**Figure 2:**
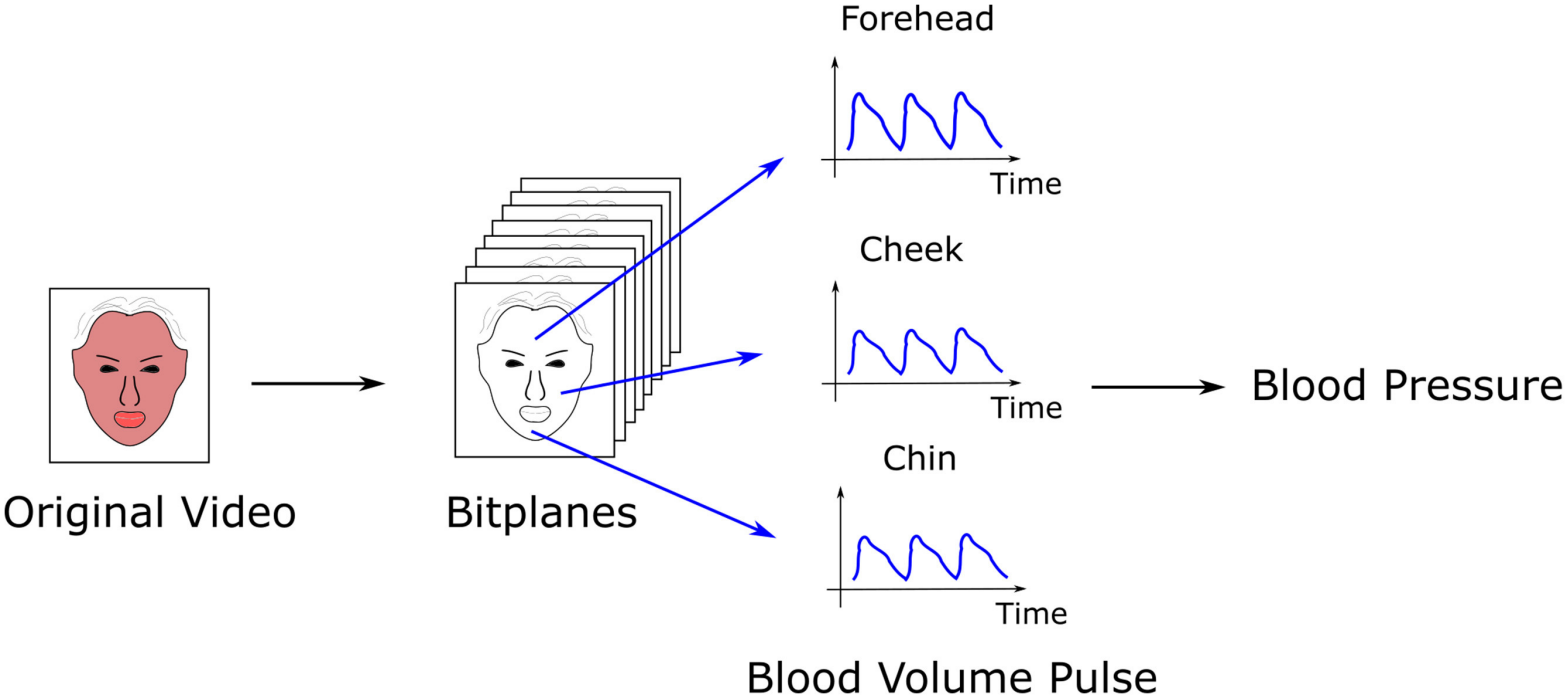
Calculation of blood pressure (BP) by transdermal optical imaging (TOI). A machine learning algorithm selects the bitplanes from a video with the highest signal for hemoglobin, from which the blood volume pulse is extracted from multiple facial regions (17 regions total). Features of the pulse are input into a multilayer perceptron to calculate BP.

A study by Luo et al. (36) with over 1,300 normo-tensive subjects demonstrated feasibility of non-contact BP measurement with video camera (64, 65). Each 8-bit image from the three color channels contains 8 bitplanes, with each pixel in a bitplane 0 or 1. A machine learning algorithm was trained to select bitplanes corresponding to hemodynamic changes. This technique has demonstrated successful calculation of heart rate and heart rate variability, stress, facial blood flow, BP, and flow responses to stimuli (36, 66, 67)(see Figure 2).

To calculate BP, transdermal blood flow data was acquired from 17 facial regions with an Apple iPhone 6. The subject was seated, with back straight and feet on the ground. Data acquisition occurred over 2-min. One hundred and twenty six features were extracted from the videos relating to pulse characteristics, such as shape, amplitude, and heart rate. An additional 29 “meta features” were selected to normalize for variation in imaging conditions across the three channels, and to account for ambient room temperature and demographic characteristics. Principal component analysis (PCA) (68) reduced data dimensions, producing 30 eigenvectors that were input into a multi-layer perceptron to calculate BP. Accuracy was approximately 95%, with an error bias of 0.39 ± 7.30 mmHg (SP), −0.20 ± 6.00 mmHg (DP), and 0.52 ± 6.42 mmHg (pulse pressure).

#### Mathematical and Optical Modeling

Adachi et al. (3) developed a mathematical model to determine contributions from hemoglobin, melanin, and light shadowing on video signal. Hemoglobin signal (PPG waveform) was extracted through removal of the melanin and lighting effects based on knowledge of the melanin light absorption spectrum and camera spectral sensitivity. Features based on waveform shape and PTT were obtained from the extracted PPG waveforms, and used to predict BP. Following recording of data for 30 s, BP was predicted under conditions of movement vs. no body movement. Without body movement, the mean prediction error from 10 subjects for SP was −1.0 ± 5.6 mmHg; with body movement, the prediction error was −0.1 ± 12.2 mmHg. Prediction error for DP was not included.

Fukunishi et al. (69) applied a model of light travel through the skin to extract the hemoglobin signal and blood volume pulse from an RGB camera. Using a high speed digital camera (capable of 2,000 fps, operated at 500 fps), with each recording set at 8.7 s, the Fukunishi model was applied by Takahashi et al. (60) to calculate PTT between forehead and chin, obtaining correlation coefficients between PTT and SP ranging from ~−0.3 to −0.8 for four subjects.

The CHROM model reduces the effect of motion and light reflected from the skin that does not possess a pulsatile or blood flow component. Fan et al. (59) and Chen et al. (53) (30 s of video, at 60 fps) (53) adapted CHROM to extract PPG signals from still videos of the face and hand, calculating PTT as the time difference between peaks or the phase difference between waveforms respectively. In both cases, subjects were sitting with facing toward the camera and hand raised. In Fan et al. (59) the authors developed a solution to a problem in video PPG where the dicrotic notch is “buried” in the overall signal, causing a peak shift and mis-estimation of PTT. This was accomplished through adaptive Gaussian modeling, where the signal is modeled as a sum of two Gaussian curves and the parameters are calculated through least squares minimization. A similar algorithm to CHROM, plane orthogonal to surface (POS), (43) was used by Fang et al. (54) to predict BP. The camera frame-rate was set to 90 fps, 15 subjects were video-imaged, with 10 videos each at 45 s per video. Similar to Chen et al. and Fan et al. above, videos of the face and hand were acquired, with PTT calculated between the cheek and radial artery in palm due to these regions providing the strongest signals.

#### Independent Component Analysis

Independent component analysis (ICA) assumes a signal is a linear mixture of underlying sources and mathematically extracts them (70). Poh et al. (71) demonstrated data from the color channels could be decomposed into three components, one corresponding to the blood volume pulse. The signal with the largest frequency component corresponding to heart rate is assumed to correspond to the blood volume pulse (72). This analysis assumes each channel contains information on hemoglobin-related fluctuations.

In a study of three healthy individuals, Secerbegovic et al. (49) applied ICA, extracting the ICA component with the largest signal at the heart rate frequency. Using PTT to estimate BP, mean absolute error for SP and mean BP were 9.48 ± 7.13 and 4.48 ± 3.29 mmHg respectively. Frame rate was 25 fps, with subject seated and videos acquired simultaneously of face (forehead) and palm, and video duration 3-min. Patil et al. (52) extracted similar features of the PPG pulse as Adachi et al. These were input into a single hidden layer neural network, obtaining average error rates of 8.4–9.62% (systolic) and 11.18–11.63% (diastolic) between afternoon and evening sessions. Subjects were permitted small head movements to simulate realistic work conditions. Oiwa et al. (56) correlated facial PPG amplitude with reference BP following ICA, obtaining a mean absolute error in the range 1.50–4.15 over eight subjects. Data was acquired over a series of 2-min resting state segments with eyes closed and 1-min cold stimulus state segment, where subjects placed their hand in a cold (14°C) water bath with eyes opened.

#### Principal Component Analysis

Most of the video signal is not attributed to blood flow fluctuations. PCA (73) calculates the main components that contribute to signal intensity variation in an image. Jain et al. (47) defined the PPG signal for each frame as the difference between the raw video data in the red channel and the main principal components. Twenty features in the time and frequency domain (6, 74) were input into a polynomial regression algorithm (75) to calculate BP. Mean absolute error was 3.72 ± 5.08 mmHg for DP and 3.90 ± 5.37 mmHg for SP. Subjects were seated still with eyes closed. Videos were acquired over 1-min. The initial and final 5-s of the videos were discarded, with the best 10-s of the remaining video processed further for analysis.

## Discussion, Perspectives, and Future Outlook

This review highlighted smartphone and video camera techniques for measuring BP. Wearables, such as watches or similar devices (76, 77), are outside the scope of the review, although they increasingly play an important role in the field of BP monitoring. Most smartphone methods for predicting BP are PPG-based. Although Peng et al. (10) attached a stethoscope to the smartphone microphone, using heart sounds only to estimate BP, such studies are relatively infrequent.

Smartphone BP monitors are potentially applicable to ambulatory BP monitoring (examining BP continuously during the day). Measurements in a doctor's office are affected by “white coat” syndrome, where patients are recorded as possessing a higher BP recording when measured clinically. Since BP varies throughout the day, improved understanding of how and why these changes occur could assist physicians in prescribing medication. Smartphones may measure pressure easily, with no additional equipment required beyond the phone or case. In contrast, cuffs may be awkward and bulky, inducing arm soreness or rashes after multiple daily uses (78). Although a smartphone may not be used at night when the user is asleep, video cameras with infrared light can monitor vital signs using similar techniques as those developed for the smartphone (79).

Contact methods have higher signal-to-noise-ratio due to the proximity of skin to the sensor and usage of light beyond ambient light (i.e., LED from phone). Additional features in the pulse wave are distinguishable in contact vs. non-contact methods, such as secondary peaks. In using machine learning algorithms to relate waveform shape to BP, these additional features are helpful in increasing the accuracy of the prediction as they may be affected by BP. Contact methods are also less dependent on motion since the finger remains pressed against the camera.

Non-contact methods possess other advantages. Contact methods are influenced by the force with which the finger is pressed against the camera. This could vary between individuals, and between trials conducted in the same individual. For waveform analysis, data from only a single region (fingertip) is acquired. Non-contact methods acquire images of multiple regions simultaneously. This enables simultaneous analysis of waveform shape and PTT. Different regions of the body could be affected differently by the sympathetic and parasympathetic nervous system, which is not accounted for through analysis of a single region. Contact methods that incorporate PTT may require additional sensors such as the microphone which produce a weak signal, or a case containing multiple sensors such as an electrocardiogram and PPG (27, 80). Oscillometry may require a special case to simultaneously measure blood volume changes and applied pressure (16), or the 3D Touch feature on the iPhone X (17), which is not available in all phone types. Non-contact methods do not face this issue since all phones are equipped with a camera.

Several published methods possess a mean error ± standard deviation within the clinically acceptable 5 ± 8 mmHg. This is not necessarily translatable to the clinic, as indicated in the trial of the AuraLife app (14). Small sample sizes of published studies do not necessarily hold for large populations. It is difficult to compare published techniques based on accuracy measures, or to predict which will be successful when applied to large populations. Studies mostly use normotensive subjects, which may reduce prediction accuracy at high and low BP. In a follow-up study of the Anura TOI-based smartphone app (81), lower BPs tended to be overpredicted, while higher BPs were underpredicted. This was attributed to more limited training data at the extreme ends of BP (81).

Many studies do not meet criteria for validating BP devices. In addition to the AAMI criteria of MAE 5 ± 8 mmHg, several other criteria are listed (82), such as: at least 85 subjects; probability of tolerable errors <10 mmHg is at least 85 %, where a tolerable error is calculated as an average of three measurements against a reference BP; reference BP measurements acquired by two observers; and recording of number of absolute BP differences within 5, 10, and 15 mmHg. The protocol must cover a sufficient time frame to ensure that as the measurement device ages, accuracy is not reduced (83). Many clinical validation protocols are tested on new models, without testing sustained accuracy over time, even though BP devices such as sphygmomanometers decline in accuracy over 18-months (84, 85). Over time, an individual may undergo physical changes in skin (i.e., aging) or changes in size, which may influence PPG extraction and BP estimation. Nevertheless, since non-contact methods should be applicable across camera types and imaging conditions, algorithms trained on data from a variety of subject types (age, sex, still vs. movement, range of skin tones and types) should be accurate for a sustainable time period.

TOI possesses advantages to other techniques for clinical translation. The sample size of Luo et al. (36) is over 1,300; 155 features over 17 ROIs relating to waveform shape, population demographics and PTT predicted BP. This is advantageous over methods with small sample size and those that only measure PTT or analyze a single region. A disadvantage of limiting measurements to PTT is the phase shift used to measure PTT partially depends on skin variability/inhomogeneity, affecting PTT accuracy (63).

Luo et al. (36) acquired images under strict conditions: normotensive, and consistent lighting and camera angle. Future studies may include a wider range of pressures to determine whether TOI may predict hypertension, and a variety of camera angles and lighting conditions.

Video-camera processing techniques other than TOI may be successful if applied to a larger population, or through optimized analysis of PPG waveform features. For example, Adachi et al. (3) only used eight features of the PPG waveform and the time difference between two pulse waves from 10 subjects as input features for learning. Due to the small sample size, it is difficult to extrapolate their success to larger populations. Other algorithms propose first classifying PPG waveforms into one of three categories (hypotensive, normotensive, or hypertensive), calculating BP according to the category to which the PPG pulse was assigned (86). This method is an improvement over traditional techniques which apply a generic algorithm to calculate BP regardless of the subject's BP range. Eulerian video magnification is a video processing technique that enhances blood flow signal (87). It has been applied to calculate PTT in videos of wrist and neck, indicating its applicability to BP measurements (88).

Future experiments may forego traditional image processing techniques. Chen and McDuff (89) developed DeepPhys, a convolutional neural network, and applied it to video frames to recover the blood volume pulse, measuring heart and breath rate. Convolutional neural network techniques may only produce a single, total blood volume pulse. TOI, however, predicts BP from multiple facial regions. This is advantageous since each region is differentially innervated, possibly influencing pressure prediction. The potential to reduce dependence on feature extraction is exemplified in the study by Baek et al. (21), who applied convolutional neural networks to PPG data without feature extraction to predict BP.

Additional possibilities are outlined in (90). This includes (A) development of techniques and models robust to “real-life” conditions such shadowing or movement; (B) application of infrared light, which acquires videos in dark conditions and may be less sensitive to variable ambient lighting; (C) development of a large publicly available dataset, where different algorithms may be applied and compared; (D) extraction of additional features beyond PTT; (E) development of a model requiring fewer calibrations.

This paper highlighted and compared the variety of methods available for measuring BP with smartphones/video cameras. It emphasizes variations in experimental design and the relevancy of these variations in developing methods for BP measurement that are efficient, easy to use, and non-invasive. This permits regular BP monitoring, contributing to early hypertension or cardiovascular disease risk detection. Possibilities for successful BP monitoring was demonstrated in early studies, such as Lamonaca et al. (5), that used the rear camera of the phone in combination with the LED to extract a strong pulse signal in the finger. Later studies included more features for analysis (18), or used convolutional neural networks to predict BP without waveform feature extraction (21). In parallel, non-contact methods were developed that overcame deficiencies of contact methods, such as BP prediction limited to a small field of view (fingertip). Non-contact video methods initially processed data from single channels, which is affected by motion, lighting, and features not related to hemoglobin such as melanin. Recent techniques, such as TOI or optical models, have extracted the hemoglobin signal specifically and are less sensitive to artifacts from non-hemoglobin sources.

Smartphone and video BP measurements will likely become more common. Compared to cuff-based techniques, they are cost-effective and convenient. Using a single video, BP may be combined with heart rate detection and stress assessment (66), blood oxygen saturation (23), and blood flow. This technology can be developed for improved digital health consultations to assess a number of health conditions. For example, measurement of the multiple parameters described above currently requires a visit to a medical health professional. This is time consuming, and may necessitate meetings with multiple health professionals.

As methods and techniques for processing video images advances, it is foreseeable a video consultation over Zoom can relay to a doctor/nurse a patient's vitals (heart rate, BP, oxygen saturation, respiratory rate, etc.) and relate this information to stroke risk or susceptibility to cardiovascular disease. This information would be provided in real-time and reduce the need for manual measurements by a medical professional. In addition, through continuous and regular daily monitoring of their own vitals privately with a smartphone, patients can be alerted *via* algorithms whether further treatment is necessary. Ambulatory BP monitoring (BP monitoring at regular intervals during day, such as *via* a video camera) may detect abnormal variations in BP not detectable with a single BP measurement session at a doctor's office. Furthermore, ambulatory BP has been demonstrated to correlate more strongly with organ damage caused by hypertension than BP measurements conducted in a clinical setting (91). Data acquired *via* smartphone may be automatically directed to a health care team for further discussions and medical decisions. Such technology can be extended to blood sugar measurements for diabetes patients, and other information related to cholesterol, fats, and hemoglobin (92). Video technology could be on the cusp of a future where a patient's home is transformed into a “smartphone-based doctor's office” where numerous cardiovascular or blood-related metrics are assessed that would previously require expertise and communication across multiple health divisions.

Overall, smartphones and video cameras will provide a more complete and earlier assessment of cardiovascular physiology, helping to prevent stroke and blood vessel-related disorders.

## Author's Note

This review discusses video camera methods for measuring blood pressure. During Covid, there has been an increase in the number of virtual consultations with patients. Thus, there is increased interest in developing technologies that will allow patients to monitor vital signs from home. This prevents unnecessary additional trips to doctors, provides daily information on changes in cardiovascular health, and may help detect signs leading to stroke or disease. In 2019, our lab published a paper in Circulation: Cardiovascular Imaging: “Smartphone-based blood pressure measurement using transdermal optical imaging technology” by Luo et al. In a study of over 1300 subjects, we demonstrated accurate blood pressure prediction *via* video camera. This was achieved through an imaging technology, transdermal optical imaging (TOI), that uses machine learning to extract a cardiovascular pulse signal from facial videos. As discussed in our review, there are numerous additional smartphone technologies beyond TOI that can also be used. Our review compares the different techniques and their technical aspects such as image processing and data analysis. The review concludes with the future of video camera blood pressure measurement, and how it can be combined with measurement of other metrics for a more complete assessment of cardiovascular health.

## Author Contributions

All authors listed have made a substantial, direct and intellectual contribution to the work, and approved it for publication.

## Funding

This work was supported by Natural Sciences and Engineering Research Council of Canada to Z-PF (RGPIN-2014-0671).

## Conflict of Interest

KL reports a patent, system, and method for contactless blood pressure determination (US 10, 376, 192 B2), licensed to Nuralogix Corporation. The remaining authors declare that the research was conducted in the absence of any commercial or financial relationships that could be construed as a potential conflict of interest.

## Publisher's Note

All claims expressed in this article are solely those of the authors and do not necessarily represent those of their affiliated organizations, or those of the publisher, the editors and the reviewers. Any product that may be evaluated in this article, or claim that may be made by its manufacturer, is not guaranteed or endorsed by the publisher.
